# Arterial roads and area socioeconomic status are predictors of fast food restaurant density in King County, WA

**DOI:** 10.1186/1479-5868-6-46

**Published:** 2009-07-24

**Authors:** Philip M Hurvitz, Anne V Moudon, Colin D Rehm, Laura C Streichert, Adam Drewnowski

**Affiliations:** 1 Department of Urban Design and Planning, University of Washington, Seattle, WA, USA; 2 Snohomish Health District, Snohomish County, WA, USA; 3 University of Washington Center for Obesity Research, University of Washington, Seattle, WA, USA

## Abstract

**Background:**

Fast food restaurants reportedly target specific populations by locating in lower-income and in minority neighborhoods. Physical proximity to fast food restaurants has been associated with higher obesity rates.

**Objective:**

To examine possible associations, at the census tract level, between area demographics, arterial road density, and fast food restaurant density in King County, WA, USA.

**Methods:**

Data on median household incomes, property values, and race/ethnicity were obtained from King County and from US Census data. Fast food restaurant addresses were obtained from Public Health-Seattle & King County and were geocoded. Fast food density was expressed per tract unit area and per capita. Arterial road density was a measure of vehicular and pedestrian access. Multivariate logistic regression models containing both socioeconomic status and road density were used in data analyses.

**Results:**

Over one half (53.1%) of King County census tracts had at least one fast food restaurant. Mean network distance from dwelling units to a fast food restaurant countywide was 1.40 km, and 1.07 km for census tracts containing at least one fast food restaurant. Fast food restaurant density was significantly associated in regression models with low median household income (p < 0.001) and high arterial road density (p < 0.001) but not with percent of residents who were nonwhite.

**Conclusion:**

No significant association was observed between census tract minority status and fast food density in King County. Although restaurant density was linked to low household incomes, that effect was attenuated by arterial road density. Fast food restaurants in King County are more likely to be located in lower income neighborhoods and higher traffic areas.

## Introduction

Obesity rates among US children and adults follow a sharp socioeconomic gradient [1]. Limited access to healthy foods by lower income groups may be at the root of the obesity epidemic [2]. Public health and policy interventions are increasingly directed at improving the food supply [3] and the built environment [4-7].

Fast food restaurants (FFRs) are said to represent an environmental risk factor for obesity [8]. The density of FFRs was reported to be higher in lower-income areas [9]. FFRs density was higher in lower income and predominantly African-American neighborhoods in New Orleans [10] and in New York City [11]. Poor areas in Melbourne, Australia were found to have 2.5 times the density of FFRs than the wealthiest areas [12]. In Scotland and England, the density of FFRs per-capita was also linked to area deprivation [13]. Indeed, mere physical proximity to FFRs in the US was said to be predictive of lower-quality diets [8,14,15].

FFRs were also reported to cluster around schools [16,17]. A Chicago-based study found that areas within 1.5 km of schools had 3–4 times as many outlets than might be expected if FFRs were uniformly distributed throughout the city [18]. Suggesting that the fast food industry targeted schoolchildren, the authors argued for zoning restrictions, specifying minimum distances between FFR and schools or playgrounds [18].

Theory and practice from geography and urban planning suggest that businesses are not uniformly distributed through urban space. Those that depend on walk-in or drive-in customers typically locate in high traffic areas [19], as indexed by measures of population density and availability of major transportation networks [20]. For FFR siting, relevant data include proximity to the intersections of road or streets with high traffic (pedestrian, transit, or automobile) volumes. Outside of densely populated urban areas, it is exposure to automobile traffic that will guide the site selection of most FFRs [20-22].

Observational studies of food environments and health [e.g., [6,7,23]] have rarely taken urban planning, zoning, and transportation issues into account [24]. Zoning regulations ensure that businesses can only locate in areas zoned for their activities and their distribution is not uniform. In New York City, the density of national fast food chains was highest in commercially zoned areas, as distinct from areas designated as residential and manufacturing zones [11].

This study examined potential associations between demographic factors, a novel transportation variable and FFR density in King County at the census tract level. The hypothesis was that arterial road density would be a powerful predictor of FFR location along with area socioeconomic status. Arterial density, based on length of roadway per census tract unit area, was a novel metric of the built environment.

## Methods

### Data Sources and Measures

#### Census Tracts

King County has 373 census tracts with a mean area of 14.8 square km per tract. Socioeconomic status was captured by median household income per tract and percent of residents who were nonwhite. Median household income and race at the census tract level were obtained from the 2000 US Census SF3 and tract boundaries were obtained from TIGER/Line data sets. Because of the low proportions of many racial subgroups in the county, race was aggregated into a single variable representing the percent of nonwhite residents per tract. Given the non-normal distribution of percent of persons living below poverty, median household income was used as a measure of area SES.

#### Residential Properties

A data set of all properties in the County was obtained from King County GIS Services; this was queried to select only residential parcels (for measuring accessibility of residential dwelling units to FFRs). Residential parcel polygons were converted to centroid points; parcels for properties containing multiple residential units were converted to multiple records to represent individual dwelling units (for measurement of per-dwelling unit distance to the nearest FFR).

Due to the large number of point features (620 FFRs and 767,274 residential units), the standard vector-based method of measuring network distance between dwelling unit and FFR points was impractical. An alternative method was used; FFR locations were "snapped" to the street network, and GIS "costdistance" modeling was used to obtain a raster measuring the roadway network distance to the closest FFR. The raster was converted to set of points at 10 ft spacing. A spatial join between residential unit points and the costdistance points tagged each residential unit record with its distance to the closest FFR. The tract-level mean distance from residential parcels to the closest FFR was calculated from individual distances.

#### Fast Food Restaurants

A list of names and addresses of all establishments licensed to prepare, sell, and serve food in King County was provided by Public Health – Seattle & King County, the local public health agency. All establishment addresses were geocoded by matching against King County GIS parcel data, which were encoded with street address.

FFRs were defined as foodservice establishments at which food was paid for at the time of ordering and prior to consumption, that did not provide table service, and that were either individual restaurants or belonged to national or local chains. A total of 620 restaurants met the FFR classification criteria, including large national chains (e.g., McDonald's, KFC) local chains (e.g., Dick's Drive-In, Kidd Valley) and individual restaurants (e.g., Burger Hut, Small Frye's). Chain or store names were used to identify national and local chain FFRs; those food sources that were not part of chains were reviewed individually by inferring from establishment name (e.g., "Hot Dog Stop," "Zest Fast Food"), reviewing company web sites, checking menus, and/or customer descriptions and reviews with online data sources including Google, Yahoo, and Yelp. Based on establishment name, FFRs were also classified by type of food sold: hamburger (220), sandwich (203), taco (122), fried chicken (29), hot dog (25); the remainder were Asian, seafood, and BBQ restaurants (a complete list is available on request).

This detailed procedure was necessary because no single commonly accepted data source type or definition of FFRs exists. Previous studies have used data obtained from telephone directories [10,25,26], commercial business data vendors [27,28], or local public health agencies [17,29]. In other studies, FFRs were classified by industrial business codes [27,29], method of payment or service [25,30], or being part of a local or national chain [10,31].

An "identity" function in the GIS assigned each FFR to the tract identifier in which the FFR point was located. Each FFR was allocated to the single census tract containing its coordinate location, even though FFR points were typically located on arterial streets and might have served 2 or even 4 census tracts if located at intersections. Access to FFRs, the dependent variable, was represented by the count of FFRs per census tract, transformed to restaurants per km^2 ^and restaurants per capita within the regression models. Network distance from residential units to the closest FFR was calculated as a measure of pedestrian or car access.

#### Arterial density metric

Arterial density was used to represent transportation infrastructure. Arterial street data were extracted from the King County GIS street line data, in which each segment of roadway was coded by the King County Department of Transportation for road type (freeway, collector, primary, local, and minor), according to US Federal Highway Administration standards [32]. Following this standard, the arterial streets used in this study were defined as those roads bearing the most traffic (freeway, collector, primary); local and minor streets were excluded because these were less likely to be zoned to allow commercial land use. A GIS "intersect" operation was used to assign each arterial segment or topologically "clipped" sub-segment to its respective census tract identifier. Arterial density was calculated in km/km^2 ^by dividing summed length of arterial roadways by tract area.

### Analysis

Log-linear regression models tested the relative contribution of independent variables to the variation in FFR density. Negative binomial regression models were used because many census tracts contained no FFRs. In order to account for differences in size or population of tracts, an offset of log (area) and log (population) was applied within the respective generalized linear models, a standard operation for modeling exposures (rates) using count data [33]. Two regression models were used with FFR density as the dependent variable: model one, based on the independent variables income and percent of residents who were nonwhite, and model two, which added arterial density as an additional independent variable. Models were estimated separately for both area and population based density of FFRs, and for both raw and z-score standardized independent variables. Models were compared using the Akaike Information Criterion (AIC) and Bayesian Information Criterion (BIC) statistics; lower AIC and BIC values indicate improved model fit adjusting for the number of model parameters.

Geocoding and spatial analyses were performed with ArcGIS v.9.3 (ESRI, Redlands/CA, 2005). Statistical analyses were performed with R v.2.8.1 statistical software (R Development Core Team, Vienna/Austria, 2008).

## Results

King County population was predominantly white (>75%) and relatively affluent with <8% of residents living below poverty. Median household income was >$57,000 USD per year [34]. Year 2000 US census data reported a mean population of 4,657 persons per tract.

**Figure 1:**
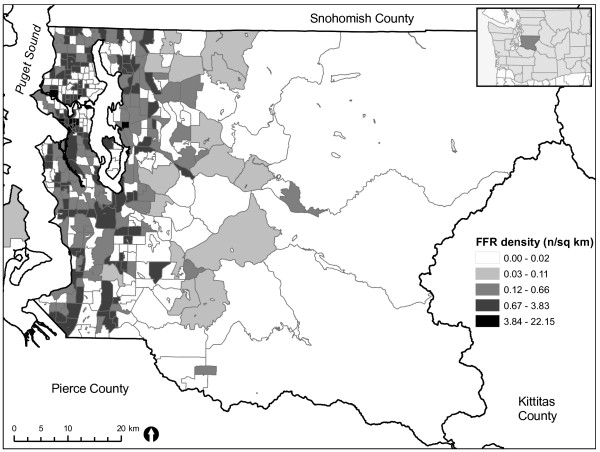
**Fast food restaurant density, King County census tracts, 2006**.

Fast food restaurant density ranged from 0 to over 22 FFRs per km^2 ^of tract (Figure 1), with the greatest densities of FFRs in the most densely populated areas. The mean network distance from all residential dwelling units in the County to at least one FFR was 1.40 km. A large number (41%) of residential dwelling units had at least one FFR within 1 km, whereas 95% had at least one FFR within 3 km.

As shown in Table 1, over one half (53%) of census tracts in the county contained at least one FFR; in those 198 tracts, the mean FFR density was 1.20 per km^2 ^of tract, and the mean network distance from residential parcels to FFRs was 1.07 km. Census tracts with FFRs generally had a higher proportion of nonwhite residents, lower median household income, and higher poverty rates.

**Table 1:** Descriptive statistics for King County, Washington State, USA

	*Countywide^a^*	*All tracts^b^* *(n = 373)*	*Tracts with fast* * food restaurants^b^* *(n = 198)*	*Tracts without fast* * food restaurants^b^* *(n = 175)*
*variable*	*mean (sd)*	*mean (sd)*	*mean (sd)*	*mean (sd)*

Total population^c^	1737034	4657 (1518)	4964 (1595)	4379 (1391)
Population density (n/sq km)^c^	315	2074 (2005)	2314 (2443)	1858 (1477)
Area (km^2^)^c, d^	5519	14.8 (95.2)	13.1 (81.8)	16.7 (108.5)
Arterial density (km/km^2^)^d^	0.77	3.45 (3.39)	4.16 (3.98)	2.62 (2.31)
% Living below poverty^c^	7.62	7.7 (6.67)	9.47 (7.32)	5.69 (5.16)
% Nonwhite^a^	24.27	23.62 (16.18)	26.50 (16.50)	20.37 (15.21)
Median household income (USD)^c^	53157	57047 (19569)	50097 (16528)	64910 (19805)
Fast food density (n/km^2^)^d^	0.11	0.81 (2.14)	1.53 (2.74)	
Fast food density (n/1000 persons)^d^	0.35	0.39 (0.82)	0.75 (1.00)	
Mean network distance from residential dwelling unit to the closest fast food restaurant (km)^d^	1.20 (.090)		1.07 (0.77)	1.88 (0.85)

^a ^summary statistics generated from non-aggregate data

^b ^summary statistics generated from tract-aggregated data

^c ^source: US Census (2000)

^d ^source: King County GIS (2007)

**Figure 2:**
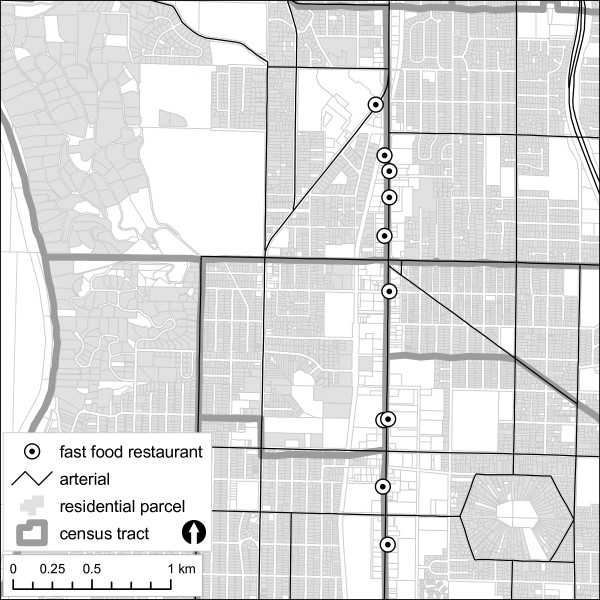
**Example of spatial arrangement of fast food restaurants, arterial streets, parcels, and census tracts in a medium-density residential neighborhood in King County, 2006**.

Figure 2 shows a typical arrangement of FFRs located along arterial streets. In all tracts, 48% of FFRs were located on arterials versus local streets; for those FFRs located on local streets, the mean distance to the closest arterial was only 49 m, effectively placing most FFRs on or very close to major roadways. Census tracts containing FFRs also had higher arterial density.

Multivariate regression models are summarized in Table 2. Using data for all 373 census tracts, model one showed that area-based FFR density was inversely associated with median household income. Each additional $1,000 decrease in median household income was associated with an increase in FFR density of 6.4% (restaurants per km^2^) and 3.9% (restaurants per capita). Percent of residents who were nonwhite was modestly and inversely associated with FFR density; a 1% decrease in nonwhite residents was associated with an increase of 1.2% and 1.09% increase in FFR density (restaurants per km^2^and per capita, respectively).

**Table 2:** Regression models for fast food density (raw and z-score standardized data), King County, Washington State, USA census tracts

	*Area-based density (fast food restaurants/tract km^2^)*				*Population-based density (fast food restaurants per tract capita)*			
**Raw data**								
**Model 1**	Coeff	Std. error	z value	Pr(>|z|)	Coeff	Std. error	z value	Pr(>|z|)
Intercept	**3.097**	0.444	6.971	**<0.001**	**-5.537**	0.389	-14.243	**<0.001**
Median hh income ($1000)	**-0.066**	0.006	-10.451	**<0.001**	**-0.040**	0.005	-7.349	**<0.001**
% Nonwhite	**-0.012**	0.006	-1.986	**0.047**	**-0.011**	0.006	-1.968	**0.049**
**Model 2**								
Intercept	**1.084**	0.469	2.310	**0.021**	**-6.366**	0.444	-14.332	**<0.001**
Median hh income ($1000)	**-0.045**	0.006	-7.145	**<0.001**	**-0.032**	0.006	-5.376	**<0.001**
% Nonwhite	-0.006	0.006	-0.97	0.332	-0.011	0.005	-1.944	0.052
Arterial density (km/km^2^)	**0.168**	0.023	7.155	**<0.001**	**0.086**	0.023	3.767	**<0.001**
**Standardized data**								
**Model 1**								
Intercept	**-0.959**	0.089	-10.774	**<0.001**	**-8.100**	0.081	-99.963	**<0.001**
Median hh income ($1000)	**-1.292**	0.124	-10.451	**<0.001**	**-0.791**	0.108	-7.349	**<0.001**
% nonwhite	**-0.198**	0.100	-1.986	**0.047**	**-0.176**	0.089	-1.968	0.049
**Model 2**								
Intercept	**-1.029**	0.083	-12.416	**<0.001**	**-8.118**	0.079	-102.344	**<0.001**
Median hh income ($1000)	**-0.879**	0.123	-7.145	**<0.001**	**-0.617**	0.115	-5.376	**<0.001**
% nonwhite	-0.089	0.092	-0.970	0.332	-0.171	0.088	-1.944	0.052
Arterial density	**0.569**	0.080	7.155	**<0.001**	**0.292**	0.077	3.767	**<0.001**

The full model (model two) illustrated that arterial road density and median household income each had an independent effect on area-based FFR density. However, arterial road density attenuated the effect of demographic variables at the census tract level. Each $1,000 decrease in median household income was associated with a 4.4% increase in FFR density (per km^2^); whereas each additional 1 km of roads per km^2 ^of tract was associated with an 18.3% increase in area-based FFR density. Minority status was no longer significant when arterial density was added to the model.

Similar results were obtained when FFR density was calculated per capita in the full model. Each $1,000 decrease in median household income was associated with a 3.1% increase in FFRs per capita, and an increase of 1 km per km^2 ^of arterial per tract was associated with a 9.0% increase in FFRs per capita. The lower values of both the AIC and BIC metrics indicated that the full model provided a better fit than the limited model, for both area- and population-based FFR densities.

Standardized coefficients were calculated to allow comparison of the relative effects of independent variables measured with different units. For both area- and population-based FFR density, median household income and arterial density were significant in the full model. In the full standardized model, the variable with greatest relative explanatory power was median household income (-0.879 and -0.617 for the different density models), followed by arterial density (0.569 and 0.292, respectively).

## Discussion

Fast food restaurant density in King County, calculated per area or per capita, was linked to lower median household income assessed at the census tract level. These findings were consistent with data from the US [3,10], the UK [13], and Australia [12]. However, the impact of area SES was attenuated when a novel transportation variable, arterial density, was included in the model. Models including this key metric of the built environment were more robust than those based on only income and percent of nonwhite residents.

No systematic associations between fast food density and proportion of nonwhite residents were observed. It should be noted that the strongest links between differential access to healthy foods and race/ethnicity were found in more racially diverse and segregated cities: New York City [11], Detroit [35], and New Orleans [10]. By contrast, King County is 85% white, with Asians representing the largest ethnic minority group. Here, the density of FFRs was weakly linked to the percent population that was nonwhite. Care should be taken not to over-generalize data obtained from cities with very sharp socioeconomic inequality indices to other locations or to the US population at large.

Observational studies of the built environment and health would also do well to include urban form variables, including road access and transportation [20]. Theories of retail location found in urban planning and business geography typically address transport, accessibility and the economics of land use. Access and road density are among fundamental issues in the siting of retail and restaurant facilities [20]. Fast food restaurant location can also be partially explained by accessibility and visibility from high-volume roadway [20]. Such proximity might have the effect of lowering local real estate prices, making such areas more accessible to lower income groups.

Urban planning has much to offer to public health intervention strategists and policy makers. The proposed moratoria on new FFRs in some urban areas appear to be grounded in the assumption that fast food chains predominantly serve walk-in population from the adjoining neighborhood. Yet fast food restaurants, situated on major arterial roads, may serve transient consumers from outside the immediate area, with the point of purchase located at the drive through window rather than at the main service counter. A study in Scotland [30] observed that out-of-home eating outlets were located in the city center and along arterial roads and freeways, but their density was not otherwise related to measures of neighborhood deprivation. The New York City study [11] noted that national fast food chains were most dense in commercial as opposed to residential areas.

Some limitations of the present study should be noted. First, the list of FFRs did not capture the complete picture of fast food availability, as many grocery stores, supermarkets, and gas stations sell similar products. Second, data summaries based on predefined administrative boundaries such as census tracts, block groups, or other enumeration units, are often problematic. Although census polygons are designed to minimize internal sociodemographic heterogeneity, their boundaries are often irrelevant for the grouping of other spatial phenomena, including access to shops and commercial services. Data aggregation in census tracts may have strong effects on statistical relationships, an issue known as the modifiable areal unit problem (MAUP) is described in the statistical literature [36,37]. Disaggregated measures such as point buffers [10], network distances [38], or geographically "smoothed" data such as kernel density estimates [39] will be less sensitive to MAUP effects and could be usefully applied to studies of the food environment. Third, the use of geocoded data, can introduce data inaccuracies [40]. Although the geocoding process has inherent problems, matching addresses against parcel data provides a more accurate method than using streets with address ranges, as the geocoded points are placed in the parcels they represent.

Access to fast foods in Seattle-King county, measured by simple physical proximity, was ubiquitous, but obesity rates varied dramatically by area [41]. A large proportion of households in King County (41%) had at least one FFR within 1 km. Yet adult obesity rates in King County varied from 5% to 30%, depending on residential ZIP code, and were best predicted by low property values [41]. Most (64%) public schools in Los Angeles County had at least one FFR within 800 m [17]. According to the California Center for Public Health Advocacy, childhood overweight rates within Los Angeles County varied from 8% in Manhattan Beach to 41% in Wilmington [42]. Economic, as opposed to merely physical, access to food is likely to play an important role in determining diet quality and health. A clearer understanding of who consumes fast foods, where they purchase, how often and why, as well as mode of access, would help policy makers to devise more cogent proposals to improve the quality of the American diet.

## Competing interests

AD is a member of McDonald's Global Advisory Council and public trustee of ILSI North America. The other authors declare that they have no competing interests.

## Authors' contributions

PMH conceived of the study, prepared and performed initial data analysis. AVM, CDR, and AD assisted with data analysis and statistical modeling. AVM, LCS, and AD helped draft the manuscript. All authors read and approved the final manuscript.

## Authors' information

PMH is a PhD candidate in Urban Design and Planning at the University of Washington (UW). AVM is a professor of Urban Design and Planning, Architecture, and Public Health at UW. CDR is an epidemiologist at Snohomish County, Washington State. LCS is the coordinator for the UW Center for Obesity Research (UWCOR). AD is a professor of Epidemiology, adjunct professor of Medicine, and director of the Center for Public Health Nutrition and UWCOR at UW.
